# Adult Measles Outbreak in New Jersey: The Dangers of Declining Vaccination Rates

**DOI:** 10.7759/cureus.87885

**Published:** 2025-07-14

**Authors:** Allyson E Whitsett, Yaser Salah, Kevin J Callagy, Aubin Attila, Sameh Girgis

**Affiliations:** 1 Internal Medicine, Jersey Shore University Medical Center, Neptune, USA; 2 Medicine, St. George's University School of Medicine, St. George's, GRD

**Keywords:** coryza, dry cough, maculopapular rash, measles prevention, routine immunization

## Abstract

This case highlights a rare presentation of measles in an unvaccinated adult male in New Jersey, emphasizing the risks posed by declining vaccination rates. The outbreak linked to this case illustrates the public health dangers of vaccine hesitancy and insufficient immunization coverage.

A 32-year-old male presented with five days of worsening fever, nausea, vomiting, diarrhea, and a spreading maculopapular rash. Physical examination revealed petechial lesions on the soft palate, a lacy-patterned rash, and tachycardia. Laboratory findings included thrombocytopenia, electrolyte imbalances, hyperglycemia, and elevated liver enzymes.

Serologic testing confirmed a diagnosis of measles. The patient received supportive care, but delays in isolation protocols resulted in secondary transmission to four unvaccinated contacts, including three children and one adult. Public health authorities were alerted, and outbreak containment measures were implemented.

This case underscores the critical role of vaccination in preventing measles outbreaks and the need for proactive public health strategies to address vaccine hesitancy. It serves as a reminder of the consequences of declining vaccination rates and the importance of maintaining herd immunity to protect vulnerable populations.

## Introduction

In 2000, the United States celebrated a monumental public health achievement: the eradication of measles. This milestone symbolized the effectiveness of widespread vaccination campaigns. However, recent years have seen a troubling resurgence of measles outbreaks, linked primarily to declining vaccination rates. The measles virus, a highly contagious *Morbillivirus* from the *Paramyxoviridae* family, spreads through respiratory droplets and direct contact with infected individuals. Symptoms include fever, coryza, conjunctivitis, cough, and the pathognomonic Koplik spots, followed by a maculopapular rash that begins on the face and progresses caudally. Complications, including pneumonia, encephalitis, and death, highlight the importance of prevention. The measles, mumps, and rubella (MMR) vaccine remains a cornerstone in preventing this disease.

The vaccine is available in two forms: the MMR vaccine and the Measles, Mumps, Rubella, Varicella (MMRV) vaccine. MMR is offered as M-M-R II or PRIORIX in the United States [1]. The MMRV vaccine is licensed only for children aged 12 months to 12 years, combining the MMR vaccine with the Varicella (chickenpox) vaccine. The CDC recommends two doses of either the MMR or MMRV vaccine during childhood: the first at 12-15 months, and the second at 4-6 years (the second dose of MMRV can also be given within 3 months of the first) [1]. All adolescents, adults, and the elderly without evidence of immunity may receive an additional 1-2 doses. The CDC also recommends full vaccination for those traveling internationally to endemic regions [1]. If exposed, receiving the vaccine within 72 hours of exposure may offer some protection [1]. Despite robust safety and efficacy data, vaccine hesitancy has increased, often fueled by misinformation [2].

This case report describes an unvaccinated adult in New Jersey diagnosed with measles after international travel, with subsequent local transmission. This incident underscores the urgent need for public health interventions to address vaccine hesitancy and protect vulnerable populations.

## Case presentation

A 32-year-old male presented to the emergency department with a five-day history of fever, nausea, vomiting, diarrhea, and a progressive rash. The patient had recently returned from Portugal, where he traveled through both urban and rural areas. His symptoms began with nonspecific GI complaints, followed by the development of a maculopapular rash. He denied respiratory symptoms but reported generalized weakness and chest pressure. The patient had no significant past medical history and no documentation of childhood vaccinations. He reported no known exposures to individuals with similar symptoms during his travels.

**Figure 1:**
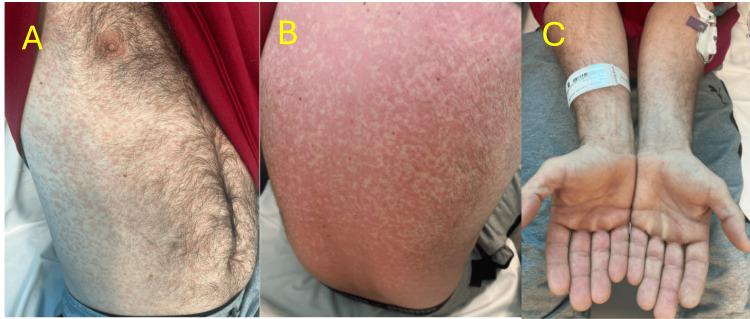
Patient’s initial presentation of maculopapular rash. A: Abdomen and chest; B: Back; C: Palms.

On examination, he was febrile (102.4°F) and tachycardic (115 bpm) but hemodynamically stable. Physical exam revealed dry mucous membranes, petechial lesions on the soft palate, and a diffuse erythematous maculopapular rash with a lacy appearance over the trunk, palms, and soles (Figure 1). While involvement of the palms and soles is atypical for measles, it was noted in this case. Lung auscultation was clear, and no lymphadenopathy was appreciated.

Initial laboratory findings included thrombocytopenia, hypokalemia, hyperglycemia, and significantly elevated liver enzymes (Table 1). Imaging studies, including a right upper quadrant ultrasound and chest X-ray, were unremarkable.

**Table 1:** Patient’s initial laboratory findings.

Laboratory Test	Result	Reference Range	Interpretation
Platelet count	106 × 10³/µL	140-450 × 10³/µL	Low (thrombocytopenia)
Potassium (K⁺)	3.2 mmol/L	3.5-5.1 mmol/L	Low (hypokalemia)
Glucose (serum)	129 mg/dL	70-99 mg/dL	Elevated (hyperglycemia)
Aspartate aminotransferase (AST)	210 U/L	0-34 U/L	Markedly elevated
Alanine aminotransferase (ALT)	418 U/L	10-49 U/L	Markedly elevated
Measles IgM antibody	Positive (1:160)	Negative	Confirmed measles infection

The initial differential diagnosis included viral exanthems, tick-borne illnesses, syphilis, and measles. Serologic testing confirmed measles with positive IgM antibodies (titer 1:160). The patient was admitted on October 7, 2024, for supportive management. He received IV fluids, electrolyte correction, antiemetics (ondansetron), and antipyretics (IV acetaminophen). Vitamin A supplementation was not administered. An infectious disease consultation was obtained. Platelet count improved from 106 × 10³/µL on admission to 166 × 10³/µL by discharge. Liver enzymes showed a rising trend: aspartate aminotransferase (AST) increased from 210 to a peak of 213 U/L, and alanine aminotransferase (ALT) decreased from 418 to 273 U/L. Despite this, the patient’s clinical status remained stable, and gastrointestinal symptoms improved.

Notably, the patient was not initially placed in isolation, raising concerns about potential nosocomial transmission. After confirmation of the measles diagnosis, airborne precautions were implemented. He was deemed medically stable and discharged from the hospital on October 10, 2024, after three days of hospitalization.

The case was reported to the New Jersey Department of Health (NJDOH) on October 10, 2024. By November 12, 2024, four additional cases of measles were identified among close contacts: one adult and three unvaccinated children. All cases were epidemiologically linked to this index patient.

## Discussion

This case underscores the critical importance of patient education and public health interventions in managing and preventing measles outbreaks. Measles is one of the most contagious infectious diseases known to humans. It is caused by the measles virus, which spreads primarily through respiratory droplets when an infected person coughs or sneezes. Alarmingly, a single individual infected with measles can transmit the virus to up to 90% of unvaccinated or non-immune individuals in close proximity [3]. The virus’s exceptional infectivity is compounded by the fact that individuals are contagious for approximately four days before and four days after the appearance of the characteristic rash, making asymptomatic transmission a major concern in outbreak settings [3].

Patient education is vital to curbing the spread of measles. Individuals must be informed about the virus’s high transmissibility and the importance of self-isolation to protect others, particularly vulnerable groups such as infants, pregnant individuals, and immunocompromised persons. This case highlights the consequences of delayed isolation, which resulted in secondary cases, and underscores the need for clear communication about the responsibility to avoid contact with others when symptomatic or suspected of having measles. Additionally, patients should be encouraged to contact healthcare providers before seeking in-person care if measles is suspected. This allows time to implement appropriate infection control measures, such as patient isolation, staff notification, and the use of personal protective equipment [4].

**Figure 2:**
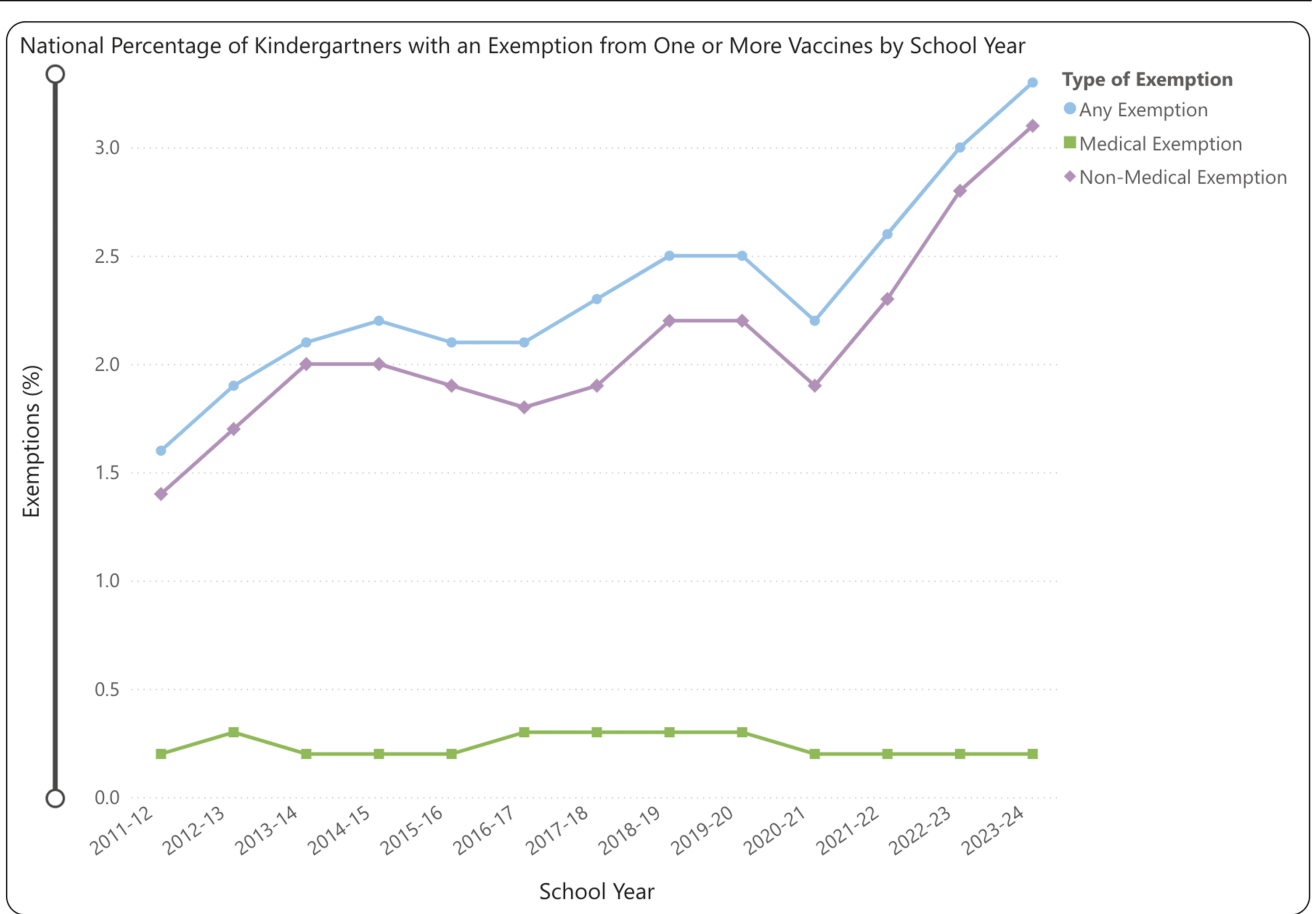
National percentage of kindergartners with an exemption from one or more vaccines by school year. Copied with permission from the U.S. CDC. Source: Reference [7].

**Figure 3:**
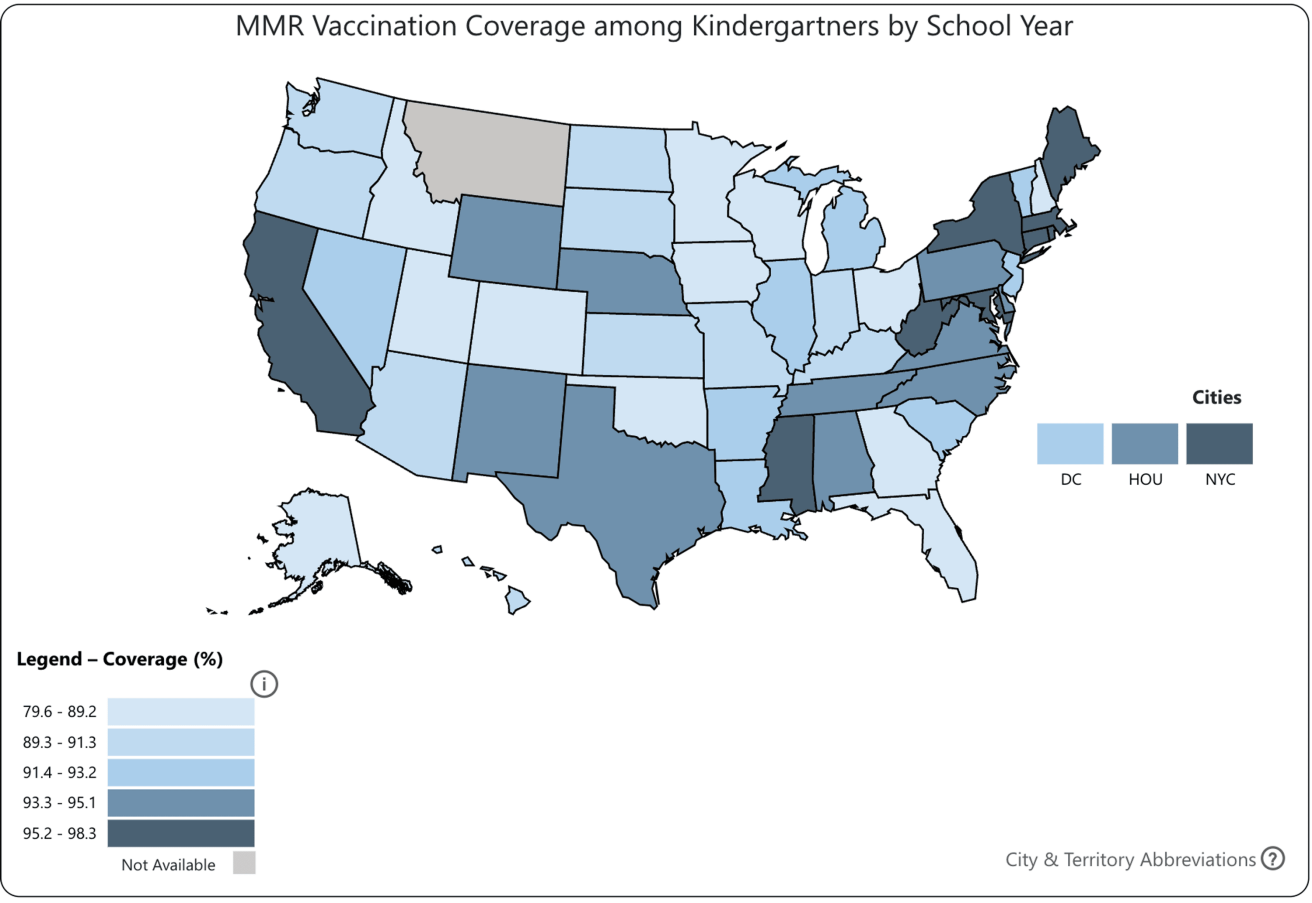
MMR vaccination coverage among kindergartners by school year. MMR: Measles, mumps, and rubella. Copied with permission from the U.S. CDC. Source: Reference [7].

Effective patient education must extend beyond individual encounters and be supported by public health campaigns to reduce vaccine hesitancy. Persistent myths about vaccine safety, such as the debunked association between the MMR vaccine and autism, continue to erode public trust in immunization programs [5]. Additional barriers, including socioeconomic inequities, limited access to care, and mistrust in healthcare institutions, compound this problem. Furthermore, nonmedical exemptions based on religious or philosophical beliefs remain a major concern, especially in states with high exemption rates (Figures 2-3) [6]. Addressing these challenges through culturally sensitive, evidence-based outreach is essential to rebuilding vaccine confidence and increasing uptake.

Global strategies have consistently emphasized the importance of achieving and maintaining high vaccination coverage to eliminate measles. The WHO recommends at least 95% coverage with two doses of the measles-containing vaccine to prevent sustained transmission and outbreaks [3]. Portugal serves as a notable example of successful implementation. With a 98% national vaccination rate, the country eliminated measles in 2015 and 2016 [8]. However, a 2017 outbreak, primarily affecting adults and resulting in a teenager’s death, demonstrated the consequences of immunity gaps, particularly in older, previously unvaccinated individuals [8].

These insights are especially relevant when considering unvaccinated adults. Such individuals may no longer be protected by herd immunity if population-level vaccination rates fall or if they travel to regions experiencing outbreaks. An unvaccinated adult with measles not only faces individual risk but also poses a public health threat, particularly to those who cannot be vaccinated due to age or medical contraindications. Targeted efforts to immunize susceptible adults remain essential to achieving and sustaining measles elimination.

In the US, declining vaccination rates amplify these risks. The national MMR coverage has recently dropped to 92.7%, falling below the 95% threshold required for herd immunity [5]. Given measles’ basic reproduction number (R₀) of 12-18, this decline poses a significant threat to outbreak control [2]. In New Jersey, the MMR vaccination rate for the 2023-2024 period was 93%, surpassing the national average. Despite this, it represents a decline from 94.3% in 2022-2023. Notably, vaccination coverage varies across neighboring counties. As of early 2025, Monmouth and Atlantic Counties each report a rate of 88%, while Ocean County trails behind at 83% [9]. These pockets of under-vaccination increase the risk of localized outbreaks, particularly among vulnerable populations such as individuals with medical exemptions. In turn, the spread of preventable diseases places additional strain on healthcare systems and contributes to broader economic and societal burdens.

To reverse these trends, multifaceted strategies are imperative. Public health campaigns must provide evidence-based information tailored to the concerns of specific communities. Collaborations with trusted community figures, such as religious or cultural leaders, can help improve vaccine acceptance [8]. Policy interventions are also essential, including reducing non-medical exemptions and increasing access to vaccines in underserved regions. Additionally, leveraging social media and digital platforms to combat misinformation and promote accurate public health messaging can effectively shape public perception.

Ultimately, this case serves as a poignant reminder of the global implications of declining vaccination rates. The patient’s unvaccinated status, coupled with international travel, illustrates how measles can resurface and spread, even in countries with strong healthcare systems. It underscores the urgent need to close vaccination gaps through proactive education, improved accessibility, and coordinated public health responses. Strengthening vaccine coverage and public trust remains key to protecting communities and preventing future measles outbreaks.

## Conclusions

The reemergence of measles in the United States, as exemplified by this case, underscores the critical role of vaccination in preventing disease transmission and protecting public health. Declining vaccination coverage, driven by vaccine hesitancy and increased exemptions, threatens the public health gains achieved over decades. Despite the availability of safe and effective vaccines, widespread misinformation and misconceptions about vaccine safety have contributed to reduced immunization rates, putting vulnerable populations at risk.

This case illustrates the far-reaching consequences of vaccine-preventable disease outbreaks and highlights the urgency of addressing vaccine hesitancy through education, policy reforms, and community-driven solutions. While vaccines may not offer absolute immunity in every individual, they significantly reduce the risk of infection by enhancing the immune response. Moreover, widespread immunization across all age groups can substantially reduce the prevalence of preventable diseases and support their eventual elimination. Ultimately, eradicating vaccine-preventable diseases requires sustained vaccination efforts, public education, and a collective commitment to community health.
